# Characterization of putative proteins encoded by variable ORFs in white spot syndrome virus genome

**DOI:** 10.1186/s12900-019-0106-y

**Published:** 2019-04-18

**Authors:** Cayro de Macêdo Mendes, Diego Gomes Teixeira, João Paulo Matos Santos Lima, Daniel Carlos Ferreira Lanza

**Affiliations:** 1 Applied Molecular Biology Lab - LAPLIC, Department of Biochemistry, Federal University of Rio Grande do Norte, Natal, RN Brazil; 2 Postgraduate Program in Bioinformatics, Federal University of Rio Grande do Norte, Natal, RN Brazil; 3 Postgraduate Program in Biochemistry, Federal University of Rio Grande do Norte, Natal, RN Brazil

**Keywords:** ORF75, ORF94, ORF125, Uncharacterized proteins, VNTR regions

## Abstract

**Background:**

White Spot Syndrome Virus (WSSV) is an enveloped double-stranded DNA virus which causes mortality of several species of shrimp, being considered one of the main pathogens that affects global shrimp farming. This virus presents a complex genome of ~ 300 kb and viral isolates that present genomes with great identity. Despite this conservation, some variable regions in the WSSV genome occur in coding regions, and these putative proteins may have some relationship with viral adaptation and virulence mechanisms. Until now, the functions of these proteins were little studied. In this work, sequences and putative proteins encoded by WSSV variable regions were characterized in silico.

**Results:**

The in silico approach enabled determining the variability of some sequences, as well as the identification of some domains resembling the Formin homology 2, RNA recognition motif, Xeroderma pigmentosum group D repair helicase, Hemagglutinin and Ankyrin motif. The information obtained from the sequences and the analysis of secondary and tertiary structure models allow to infer that some of these proteins possibly have functions related to protein modulation/degradation, intracellular transport, recombination and endosome fusion events.

**Conclusions:**

The bioinformatics approaches were efficient in generating three-dimensional models and to identify domains, thereby enabling to propose possible functions for the putative polypeptides produced by the ORFs wsv129, wsv178, wsv249, wsv463a, wsv477, wsv479, wsv492, and wsv497.

**Electronic supplementary material:**

The online version of this article (10.1186/s12900-019-0106-y) contains supplementary material, which is available to authorized users.

## Background

White Spot Syndrome Virus (WSSV) is an enveloped double-stranded DNA virus recognized for its great impact on global shrimp farming and for the complexity of its ~ 300 kb genome [1]. To date, little is known about the function of most of the ~ 184 WSSV predicted proteins since they have no homology with known sequences in the repositories [2]. Although most of these proteins present high identity among different WSSV isolates, variations are present in WSSV genome coding regions, including two genomic deletions occurring between ORFs wsv461/wsv464 (14/15) and ORFs wsv77/wsv502 (23/24), and a variable number of tandem repeats (VNTRs) occurring within wsv129 (ORF75), wsv178 (ORF94) and wsv249 (ORF125) [3]. These variable regions have been used as molecular markers to identify viral variants [4–7]. Some studies have already indicated that these variable regions may have some relationship with the viral evolution and infection phenotype [8, 9], however there are still no direct correlations between the function of these putative products and virulence, mainly due to the lack of information about the functions of these proteins.

Computational tools have proven to be efficient for functionally characterizing proteins at low cost in a shorter time, thus enabling the analysis of some targets which cannot be evaluated in vitro, such as membrane proteins [10, 11] or proteins involved in viral infection mechanisms [12–15]. In this work, the putative proteins encoded by variable regions in the WSSV genome were structurally and functionally characterized using bioinformatics tools, and possible functions for these proteins were inferred.

## Methods

Available nucleotide sequences corresponding to the variable regions from WSSV different isolates were retrieved from GenBank and subjected to a multiple alignment through MAFFT [16], and some adjustments were made by manual editing. Repeat units of each isolate were annotated using Geneious version 11.0.3 [17] and aligned against a reference sequence for annotation of polymorphism sites. Polymorphism sites were visualized by WebLogo [18].

Remote homologies between protein sequences were identified using BLAST tools (BLASTp, PSIBLAST and PHIBLAST) against non-redundant databases [19, 20]. Searches based on Hidden Markov Model profiles were also performed through JACKHMMER, hmmscan, HHBlits and HHPred [21–23].

Protein sequences were submitted in an iterative search to generate tertiary structure models using HHBlits (2–4 iterations) against the Uniclust30 database [24]. The best hits were selected from the generated output and submitted on HHPred against the PDB70 database. HHPred’s best hits were used as templates to generate the structural models. The tertiary structure was modeled with PHYRE2, ITASSER, Swiss-Model and Modeller [25–28]. All models were based on the sequences of China 01 isolate (KT995472.1).

Secondary structures and threading predictions were also generated using PSIPRED and pGenThreader, respectively [29, 30]. The obtained models were evaluated by Molprobity [31] evaluating the parameters clashscore, hydrogen bonds, van der walls contacts, geometry, rotamers, Cβ deviations and *cis*-peptides. Ramachandran plots were generated by pyRAMA 2.0 [32]. All images from the models were generated by Chimera [33]. The validation of the structural models was performed through Verify3D which evaluates the compatibility of a three-dimensional model based on the 3D-1D scores that consists of the statistical preference of each of the amino acid residues that make up the model.

Structures used as templates for protein modeling were obtained from the Protein Data Bank (PDB): U1 small nuclear ribonucleoprotein (U1 snRNP, PDB: 4PKD), XPD repair helicase of *Thermoplasma acidophilum* (PDB: 4A15), Influenza hemagglutinin HA2 subunit (PDB: 1QU1), Ankyrin Repeat (PDB: 4HDB), RNF4 RING (PDB: 4AP4), Formin mDia1 Structure (PDB: 3OBV). Disordered regions, coiled coils, transmembrane regions and signal peptides were predicted using Foldindex, Coils, TMHMM and SignalP [34–36], respectively. SMART, CDD, ScanProsite and Eukaryotic Linear Motif (ELM) were used to detect conserved domains, patterns and motifs [37–40].

## Results

Only characterization results with highest confidence levels based on the evaluation of the protein models are presented in this section. The pipelines that presented the best results for each model generated in this study are summarized in Table 1. The validation of the models was also performed using Verify3D. These results are presented in the Additional file 1.

**Table 1:** Pipelines used to generate each of the models presented in this study. The numbers represent the sequence in which each software was used

Model	HHBLITS	HHPRED	Modeller	Swiss-model	Phyre2	ITASSER	PSIPRED
wsv249 (ANK)	1	2	–	3	–	–	–
wsv249 (RING)	1	2	–	3	–	–	–
wsv463a	1	2	3	–	–	–	–
wsv477	1	2	3	–	–	–	–
wsv479	–	–	–	2	–	–	1
wsv492	–	1	–	2	–	–	–
wsv497	–	–	–	2	–	–	1

### Characterization of some ORFs which occur in wsv461/wsv464 and wsv477/wsv502 clusters

**Fig. 1:**
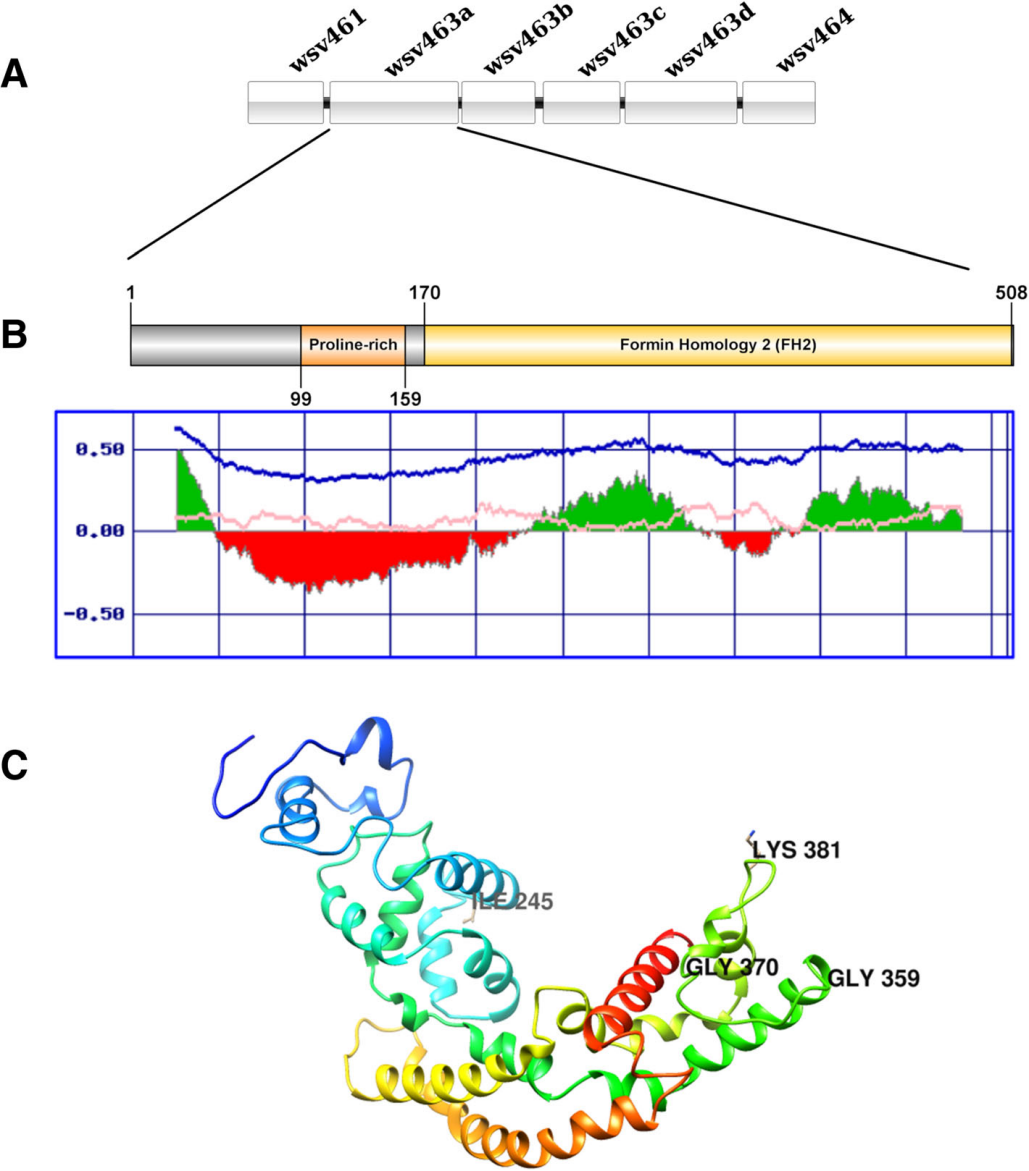
Structural analysis of the putative protein encoded by ORF wsv463a (ORF14/15). **a** Schematic representation of wsv461/wsv464 cluster. **b** Schematic representation of the putative protein encoded by wsv463a, FH1 domain corresponding to the proline rich region (orange) and Formin homology 2 domain are shown in yellow. The graph below the scheme indicates the folded (green) and unfolded (red) regions predicted by FoldIndex, the x-axis indicates the amino acid positions and the y-axis represents the probability scores. **c** Three-dimensional model of the wsv463a sequence corresponding to Formin Homology 2 domain. Conserved subdomains (Lasso, Linker, Knob, Coiled Coil and Post) and amino acids of the typical FH2 domain are indicated

The alignment of sequences corresponding to wsv461/wsv464 and wsv477/wsv502 clusters revealed insertions of approximately 5Kb and 13Kb, respectively. The wsv461/wsv464 cluster contains up to 6 ORFs (wsv461, wsv463a, wsv463b, wsv463c, wsv463d, wsv464), as detailed in Fig. 1a and Table 2. This cluster is truncated in most isolates, lacking these 6 ORFs. The insertions of the WSSV-CN02 and WSSV-TW isolates have wsv461 and wsv463 linked as a single coding region.

**Table 2:** Characterization of sequences corresponding to ORF wsv461/wsv464 (ORF14/15) cluster obtained from different WSSV isolates

Accession Number	Description (Country)	Insertion lentgh (bp)	Number of putative ORFs (including wsv461 and wsv464)	Putative ORFs
AF440570.1	Taiwan	506	2	(wsv461 f, wsv464)
AF332093.3	China	257	3	(wsv461*, wsv463 f,wsv464)
AF369029.2	Thailand	–	2	(wsv461, wsv464*)
JX515788.1	South Korea	–	1	(wsv464)
KR083866.1	Egypt	257	3	(wsv461*, wsv463 f,wsv464)
KT995472.1	China 01	4999	6	(wsv461, wsv463a, wsv463b, wsv463c, wsv463d,wsv464)
KT995470.1	China 02	506	2	(wsv461 f, wsv464)
KT995471.1	China 03	–	2	(wsv461*, wsv464*)
KY827813.1	China 04	–	2	(wsv461*, wsv464*)
MF784752.1	Brazil	–	2	(wsv461, wsv464*)
KR083844.1	Egypt 09	4894	6	(wsv461, wsv463a, wsv463b, wsv463c, wsv463d,wsv464)
KF771904.1	USA Texas	4894	6	(wsv461, wsv463a, wsv463b, wsv463c, wsv463d,wsv464)
AY753327.1	Thailand 96 II	5025	7	(wsv461, wsv463a t, wsv463b, wsv463c, wsv463d,wsv464)
KR083865.1	Egypt 14	137	2	(wsv461*, wsv464*)
KF771899.1	USA DC97	137	2	(wsv461*, wsv464*)
KR083846.1	EGYPT 11	1136	2	(wsv461*, wsv464*)
KF771902.1	USA Texas 2	1136	2	(wsv461*, wsv464*)
KX501221.1	Thailand 12	–	2	(wsv461*, wsv464*)
EU327501.1	India 05	–	2	(wsv461*, wsv464*)
MG702567.1	INDIA AP4RU	–	2	(wsv461*, wsv464*)
HQ257380.1	Mexico Mx-F	335	2	(wsv461*, wsv464*)
HQ257382.1	Mexico Mx-C	335	2	(wsv461*, wsv464*)
HQ257383.1	Mexico Mx-G	335	2	(wsv461*, wsv464*)
MF768985.1	Australia	171	2	(wsv461*, wsv464*)
KX501220.1	Thailand TH-14	–	2	(wsv461*, wsv464*)
KU746817.1	China FCG2–14	–	2	(wsv461*, wsv464*)
EF468499.1	India IN-07	335	2	(wsv461*, wsv464*)

*Partial sequences

f Fusion of two sequences

t Two ORFS formed in wsv463a (occurs exclusively on WSSV TH-96-II)

**Fig. 2:**
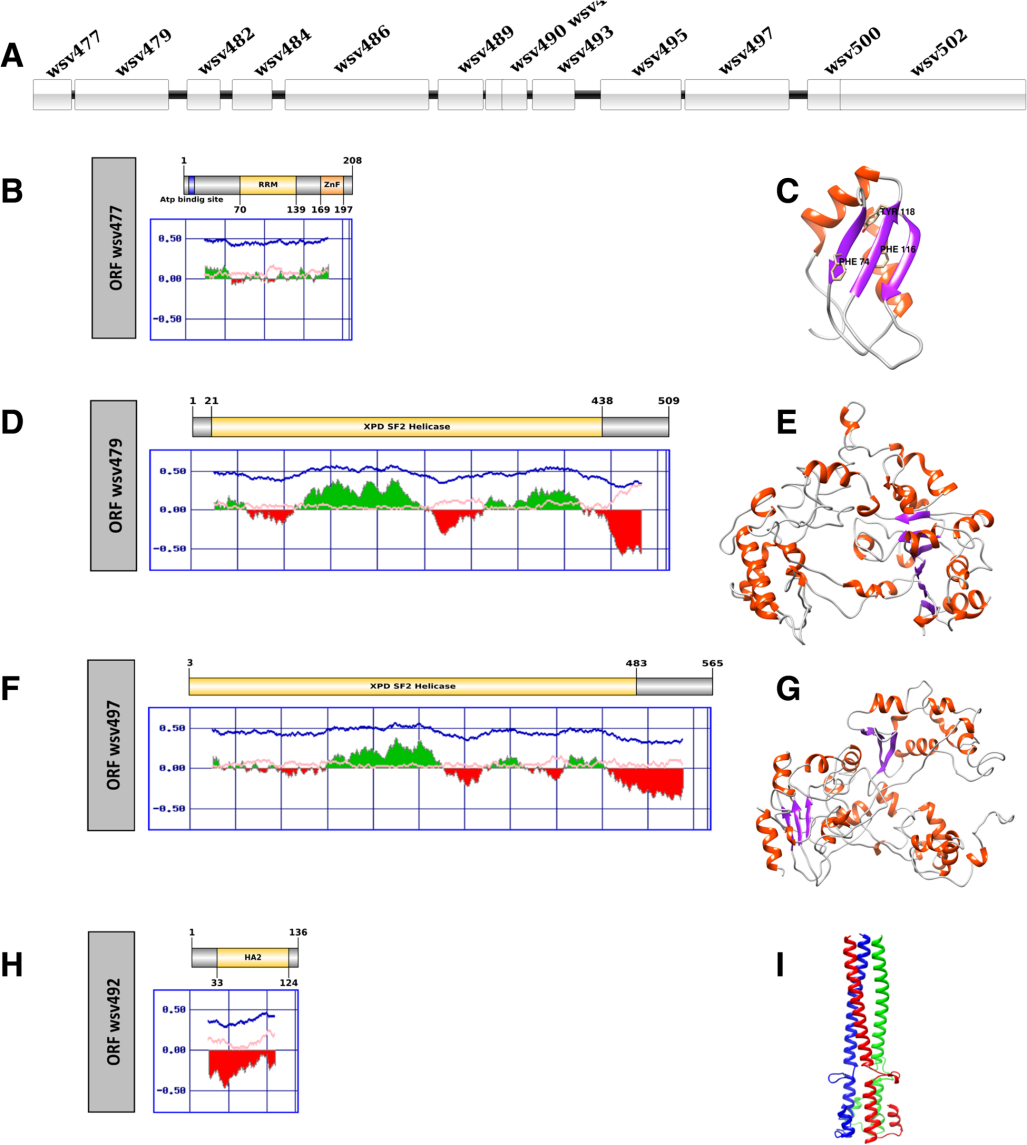
Structural analysis of the proteins encoded by wsv477/wsv502 cluster. **a** Schematic representation of wsv477/wsv502 cluster. **b**, **d**, **f**, **h** Schematic representation of putative proteins encoded by ORFs wsv477, wsv479, wsv497 and wsv492, respectively. Zinc-finger domain of the product encoded by ORFwsv477 is shown in orange and the other major domains of each protein are highlighted in yellow in each respective scheme. Graphs below each scheme indicate the folded (green) and unfolded (red) regions predicted by FoldIndex. In each graph, the x-axis indicates the amino acid positions and the y-axis represents the probability scores. **c**, **e**, **g**, **i** Three-dimensional models of the major domains identified in each predicted protein. The main conserved amino acids are presented in the RRM model (**c**) and the homotrimeric helices are highlighted in different colors in HA2 (I)

The number of putative coding regions in wsv477/wsv502 cluster is higher comprising 13 ORFs, (wsv477, wsv479, wsv482, wsv484, wsv486, wsv489, wsv490, wsv492, wsv493, wsv495, wsv497, wsv500, wsv502), as detailed in Fig. 2a and Table 3. The characterization results of each coding region for both clusters is presented below.

**Table 3:** Characterization of sequences corresponding to ORF wsv477/wsv502 (ORF23/24) cluster, obtained from different WSSV isolates

Accession Number	Description| (Country)	Insertion lentgh (bp)	Number of putative ORFs (including wsv477 and wsv502)	Putative ORFs
AF440570.1	Taiwan	12,613	13	(wsv477, wsv479, wsv482, wsv484, wsv486, wsv489, wsv490, wsv492, wsv493, wsv495, wsv497, wsv500, wsv502)
AF332093.3	China	11,451	13	(wsv477, wsv479, wsv482, wsv484, wsv486, wsv489, wsv490, wsv492, wsv493, wsv495^a^, wsv497, wsv500, wsv502)
AF369029.2	Thailand	81	2	(wsv477, wsv502)
JX515788.1	South Korea	6959	8	(wsv477, wsv479, wsv482, wsv484, wsv486, wsv500, wsv502)
KR083866.1	Egypt	11,451	13	(wsv477, wsv479, wsv482, wsv484, wsv486, wsv489, wsv490, wsv492, wsv493, wsv495^a^, wsv497, wsv500, wsv502)
KT995472.1	China 01	12,536	13	(wsv477, wsv479, wsv482, wsv484, wsv486, wsv489, wsv490, wsv492, wsv493, wsv495^a^, wsv497, wsv500, wsv502)
KT995470.1	China 02	6718	8	(wsv477, wsv479, wsv482, wsv484, wsv486, wsv500, wsv502)
KT995471.1	China 03	1643	4	(wsv477, wsv479^a^, wsv500, wsv502)
KU216744.1	Mexico	98	2	(wsv477, wsv502)
KY827813.1	China 04	1643	4	(wsv477, wsv479^a^, wsv500, wsv502)
MG264599.1	Brazil	12,090	13	(wsv477, wsv479, wsv482, wsv484, wsv486, wsv489, wsv490, wsv492, wsv493, wsv495, wsv497, wsv500, wsv502)

^a^Partial sequences

### ORF wsv463a

The ORF wsv463a, a putative coding sequence located in wsv461/wsv464 cluster (Fig. 1a), presented a proline rich domain located in an unfolded portion of the predicted protein (positions 99–159) and a larger domain similar to the Formin Homology 2 (FH2) between positions 170–508 (Fig. 1b). The 3D model of the larger structured domain confirms the alpha-helical structure of FH2, composed by five alpha-helical subdomains (Lasso, Linker, Knob, Coiled Coil and Post) (Fig. 1c). The validation tests, main scores and the ramachandran plot corresponding to the 3D model are presented in Additional file 2.

### ORF wsv477

The ORF wsv477 is the first coding region in wsv477/wsv502 cluster (Fig. 2a). The putative product of wsv477 presents a domain homologous to a RNA Recognition Motif (RRM) in positions 70–139 (Fig. 2b), and a “Zinc-Finger” domain in its C-terminal region. The 3D model of the RRM domain revealed a tertiary structure with two alpha-helices and three beta-sheets following a β1α2β2β3α2 pattern, which corresponds to the typical RRM β1α2β2β3α2β4 structure (Fig. 2c and Additional file 3). Aromatic residues involved in RNA binding remain conserved in the central strands of the proposed protein, which are composed of Phe74 at position 2 of the β1, Tyr118 at position 5 of β3 and Phe116 position 3 of β3 (Fig. 2c).

### ORFs wsv479 and wsv497

The ORFs wsv479 and wsv497 are also located in the wsv477/wsv502 cluster (Fig. 2a). These ORFs produce putative proteins that are similar in amino acid composition, size and folding, suggesting that they have similar functions (Fig. 2d, e, f and g). It was not possible to infer any function by sequence homology. By using threading approaches it was possible to determine that the tertiary structure of these two ORFs present similar folding to Xeroderma pigmentosum group D repair helicase (XPD) (Fig. 2e, g and Additional files 4 and 5). XPD belongs to the helicase superfamily 2 and a component of transcription factor IIH (TFIIH), which is associated with Nuclear Excision Repair pathway (NER), catalyzing the opening of the double helix around the damaged site, providing access to NER factors in a ATP-dependent process [41]. The XPD helicase consists of two motor domains called HD1 and HD2, an arch domain and an iron-sulfur cluster (FeS) superimposed on HD1. The process of binding XPD to DNA is through the HDR2 domain [42].

### ORF wsv492

The wsv492 putative product demonstrated high similarity with the HA2 subunit of the hemagglutinin influenza virus (Fig. 2h, i). The 3D model shows a HA2-like subunit formed by a triple-helical chain (Fig. 2i and Additional file 6).

### Characterization of ORFs comprising VNTRs

The alignment of sequence sets for ORFs wsv129, wsv178 and wsv249 revealed that high and less variable regions are wsv129 and wsv249 respectively, considering the VNTR size and the total number of analyzed sequences (Table 4). It has been observed that repeat units (RUs) contain few polymorphic sites in all cases. Substitutions in wsv178 were observed occurring in positions 1, 36 and 48. Wsv249 has substitutions in positions 2, 9, 12, 27, 50, 53 and 61, with the last three occurring at a higher frequency. Wsv129 has two types of repeat units, the most frequent having 45 bp and a 57 bp repeat intercalating the 45 bp RUs. Indels occur more frequently in wsv178 and wsv249.

**Table 4:** Repeat units in WSSV VNTRs

VNTRs	RU length (bp)	Number of aligned sequences	Number of different sizes	Minimum number of RUs	Maximum number of RUs	Most frequent RU
wsv129 (ORF75.a)	45	39	13	2	21	11
wsv 129 (ORF75.b)	57	39	5	1	5	3, 4
wsv178 (ORF94)	54	43	12	1	18	6
wsv249 (ORF125)	69	34	5	2	7	4

### ORF wsv129

**Fig. 3:**
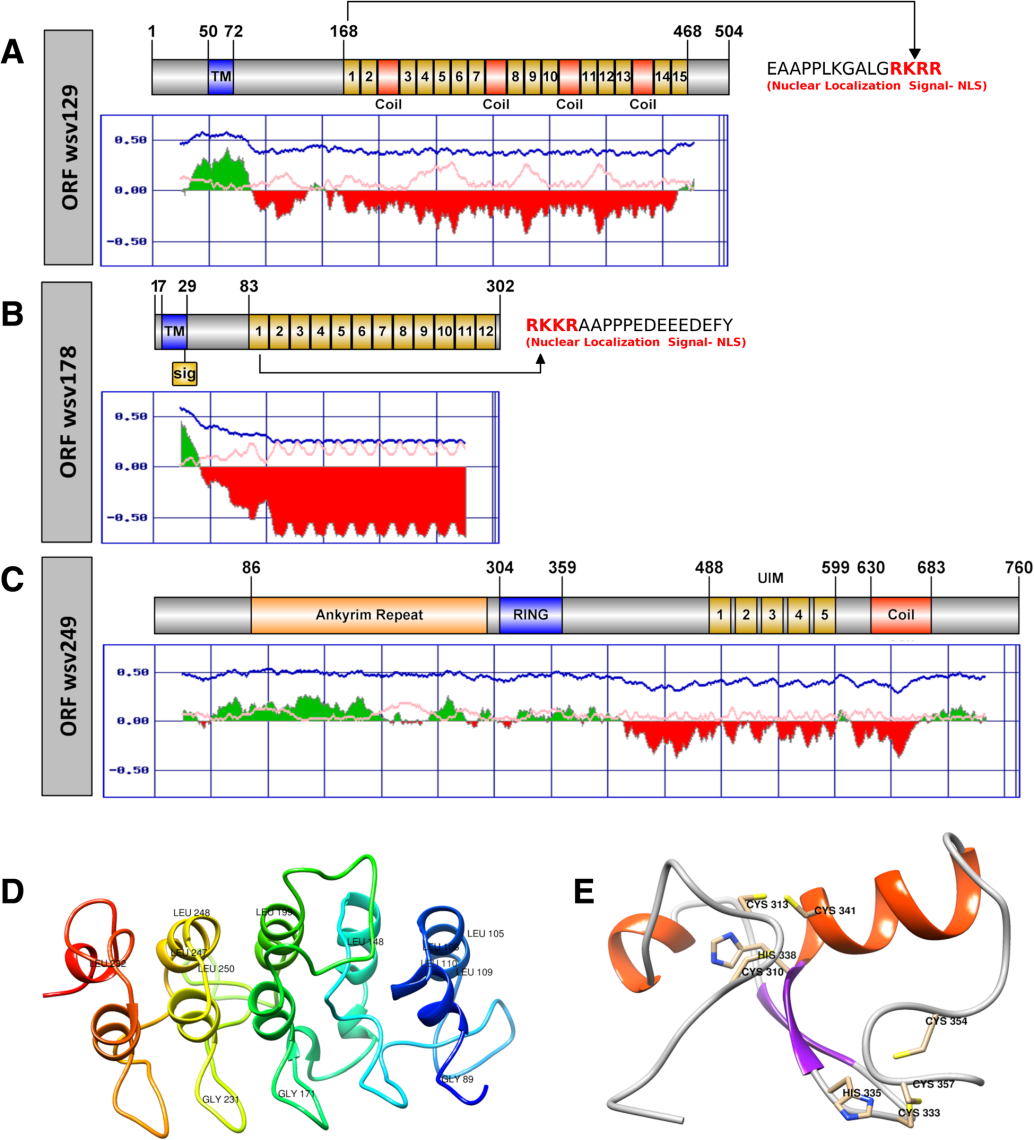
Structural analysis of the proteins encoded by ORFs containing variable number of tandem repeats. **a**, **b**, **c** Schematic representation of putative proteins encoded by wsv129 (ORF75), wsv178 (ORF94) and wsv249 (ORF125). VNTR regions are presented in yellow, the numbers correspond to repeat units (RUs). Transmembrane regions (TM) and the Ring Finger domain (RING) are highlighted in blue in each respective scheme. The nuclear localization signal of each RU is shown in red. The graphs below each scheme indicate the folded (green) and unfolded (red) regions predicted by FoldIndex. In each graph,ic the x-axis indicates the amino acid positions and the y-axis represents the probability scores. **d**, **e** Three-dimensional models of wsv249 regions corresponding to Ankyrin (**d**) and RING domains (**e**). Conserved subdomains and amino-acids are presented in each 3D-model

Secondary structure predictions revealed small transmembrane helices in the N-terminal region (positions 50–72) which coincide with a structured region of the wsv129 predicted protein (Fig. 3a). Curiously, several coiled coils coded by the 57 bp repeat units and nuclear localization signals (NLS) are predicted at the end of each repeat in VNTR region (Fig. 3a).

### ORF wsv178

Small transmembrane helices were also predicted in the N-terminal region of wsv178 putative product (positions 7–29) coinciding with the unique folded region of the protein (Fig. 3b). Curiously, a cleavage site was predicted between residues 26 and 27 which could separate the product encoded by the VNTR region from the transmembrane portion. The presence of putative nuclear localization signals was also observed at the beginning of each repeat unit.

### ORF wsv249

The product generated by wsv249 has a more structured chain compared to the other ORFs that contain VNTR regions (Fig. 3c). The characterization by remote homology and fold recognition revealed that the first 300 residues of the N-terminal region correspond to a Ankyrin repeat (ANK) motif (Fig. 3d and Additional file 7). It was also possible to identify a RING-H2 domain immediately after the ANK domain region. The 3D model of the RING-H2 domain is shown in Fig. 3e, as well as the main conserved amino acids. The validation tests of this three-dimensional model are presented in Additional file 8.

Some glycine residues (89, 171, 231) located in the second beta sheet as well as leucine residues located in second alpha-helix (105, 106, 109, 110, 148, 199, 247, 248, 250, 282) of ANK motif remain in conserved positions in the proposed model. The typical tetrapeptide TPLH was not observed in sequence due to divergence at the primary structure level.

SMART data revealed that the tandem repeat units in wsv249, despite the high E-value (due to the small sequence length), corresponding to ubiquitin-interacting motifs (UIM) which consist of 20 residues alpha-helix of a X-Ac-Ac-Ac-Ac-Φ-X-X-Ala-X-X-X-Ser-X-X-Ac-X-X-X-X consensus, where “Φ” corresponds to a hydrophobic residue, “Ac” is acidic residues and “X” can be any amino acid residue [43, 44]. Additionally, a RING-H2 was detected immediately next to the Ankyrin domain located between positions 310–357 of the protein (Fig. 3c, e), which has the Cys-X2-Cys-X(9–39)-Cys-X(1–3)-His-X(2–3)-His-X2-Cys-X (4–48)-Cys-X2-Cys pattern, where “X” comprises any amino acid.

## Discussion

### wsv461/wsv464 and wsv477/wsv502 clusters

The number of sequences with the truncated insertion is smaller in wsv477/wsv502 cluster when compared to the wsv461/wsv464. This observation is in accordance with previous results which demonstrate that some proteins encoded by wsv477/wsv502 cluster possibly have functional domains [45, 46].

### ORF wsv 463

The protein encoded by ORFwsv463 presents formin characteristics. Formins consists of a family of proteins that regulates the elongation of unbranched actin filaments which are important in many cellular processes, including formation of actin cables, cytokinetic ring, filopodia and stress fibers [47, 48]. These processes are mediated by the FH2 domain located in the C-terminal region, which forms a stable hydrophobic ring-like hemidimer and binds the ends of actin filaments protecting from capping proteins [49, 50]. Each hemidimer has conserved residues that are directly related to actin binding consisting of an Ile located in the subdomain knob and Lys located in the lasso/post interface. Mutations within these conserved sites may compromise actin nucleation activity [51]. The glycine residues at positions 359 and 370 act on dimerization, as well as Ile245 and Lys381 acting in actin-binding remain conserved in the wsv463a predicted protein (Fig. 1c).

Formins also have a FH1 domain composed of polyproline, similar to what was observed in the wsv463a protein scheme (Fig. 1b). The FH1 domain is directly related to the interaction with profilin proteins during the actin elongation. The actin monomer binding protein profilin stimulates the actin assembly through binding FH1 and FH2 domains, increasing elongation speed [51, 52].

As an essential component of cellular cytoskeleton, actin can be manipulated by viruses into the host cells in many stages of its life-cycle, including entry, motility, nuclear and assembly [53], modulating the activity of actin binding proteins. Actin filaments provide mechanical force for viral pathogens to navigate within the host cell, causing changes in cellular shape [54]. HIV is able to navigate between dendritic cells through filopodia produced by formins [54]. Formin FHOD1 together with the small GTPase Rac1 of Vaccinia virus is associated with actin tail formation, acting in an integrated way with the N-WASP-ARP2/3 pathway, thus being essential for Vaccinia virus motility and dissemination [55]. The fact that WSSV encodes formins may be related to regulation of host fibrous proteins involved in viral packaging and/or intracellular transport.

### ORF wsv 477

ORF wsv477 was previously characterized as a 624 bp immediate early gene encoding putative protein of the 208 amino acid, with an ATP/GTP binding site between positions 7–14 and a Cys2/Cys2 type Zinc-finger domain between residues 169–197 [56]. In addition, a miR-7 injection in WSSV infected shrimp could reduce the wsv477 expression and decrease the number of WSSV genome copies at 12 to 96 h post infection [57, 58]. The results presented herein corroborate that the RRM domain in wsv477 protein may be related to post transcriptional steps (splicing, pre-mRNA processing, RNA editing, translation regulation) which determine the efficiency of viral replication.

RRM can be found in all organisms, being found with greater abundance in proteins encoded by eukaryotes in multiple copies or in conjunction with other domains like “Zinc-Finger” domains of the CCCH or CCHC types, which can bind to RNA. This domain is involved in post-transcriptional events including splicing, pre-mRNA processing, RNA editing, translation regulation, and RNA degradation [59–63]. Some RRM proteins are involved in replicating RNA viruses, including heterogeneous nuclear ribonucleoprotein A1 (hnRNP A1) in Hepatitis C virus which interact with an RNA-dependent RNA-polymerase and septin 6, forming a replication complex [64]. The interface between RNA and RRM occurs through four conserved residues located in the central β1 and β3 (called RNP2 and RNP1, respectively) of the RRM, where nitrogenous bases of the RNA bind to the side chain of the localized aromatic amino acids in the β1 (position 2 of RNP2) and in β3 (position 5 of RNP1). The third aromatic residue at position β3 (position 3 of RNP1) hydrophobically interacts with the two pentose rings of each nucleotide [65, 61].

### ORFs wsv479 and wsv497

It has been previously described that wsv479 and wsv497 sequences have a conserved VP9 domain (also known as ICP11) located in the N-terminal region [66] having a ferredoxin fold which has been suggested as a DNA recognition domain. Considering this and the presented results, these proteins probably have functions related to WSSV genome processing and recombination events.

### ORF wsv492

The wsv492 putative protein probably has functions related to hemagglutinin. Hemagglutinins consist of glycoproteins which remain anchored in the viral envelope and mediates viral entry [67]. After protein synthesis, the sequence encoding hemagglutinin (HA0) undergoes a post-translational cleavage, producing the HA1 and HA2 subunits which form a homotrimeric structure. The HA1 subunit interacts with sialic acid, a monosaccharide present on the membrane surface mediating the endocytosis of the viral particle [68]. The HA2 subunit consists of a triple-helical hydrophobic structure associated with the pH-induced fusion process, a mechanism by which the virus releases from the endosome and contacts the host cell cytosol.

### ORFs comprising VNTRs

The RU profiles observed in VNTRs of ORFs wsv129, wsv178 and wsv249 coincide with those already previously described [69, 70]. Interestingly, the reading windows are maintained in even more variable VNTRs. It was not possible to obtain reliable three-dimensional models for the wsv129 and wsv178 putative products, since they have a large unstructured portion, as well as many charged amino acids.

### ORF wsv129

The prediction of a large unstructured portion rich in coiled coils in association with the transmembrane domain in the wsv129 polypeptide indicates a structural function. In fact, 18 structural proteins located in the WSSV virion, including the protein encoded by wsv129, were previously detected by proteomic analysis [71]. In this same work, a temporal analysis showed that the wsv129 product is expressed late, at least 6 h after the WSSV infection.

NLS act by directing proteins into the cell nucleus and can be subdivided into two subclasses: monopartite, formed by a group consisting of a sequence K (K/R) X (K/R), or bipartite, which are formed by two groups of basic residues separated by a 10–12 amino acid linker which may vary [72]. The transport of macromolecules through the nucleus occurs through a nucleoporin protein complex called the nuclear pore complex (NPC). Proteins above 40 kDa require a specific signal which will allow it to interact with carrier proteins that will facilitate its entry into the nucleus [73–75].

The binding of the carrier protein of the NLS is important for the release of the viral molecules into the nucleus. DNA viruses that infect animals replicate in the nucleus of the host cell. To drive the entry of the viral genome into the nucleus, large viruses normally release their DNA associated with structural proteins which are associated with nuclear localization signals [76].

Viral proteins containing NLS signals in tandem were not found in the literature. Since the mechanism of WSSV entry into the host cell nucleus is unknown, this may be an indication of a new type of entry mechanism which needs to be better investigated. On the other hand, it is not possible to rule out the possibility that these NLS signals are artifacts of the software analysis.

### ORF wsv178

The structural data presented herein indicates that wsv129 and wsv178 are related to similar functions, as previously suggested [77]. These proteins may also have some adaptive function, considering that these two VNTR are the most variable, even among isolates from the same region.

### ORF wsv249

Ankyrin repeat (ANK) is a motif composed of about 33 amino acid residues which are important in the modulation of several cell pathways mediating specific protein-protein interactions; most of the protein sequences that exhibit these motifs usually consist of transcriptional regulators, modulators of cellular development and differentiation [78]. The ANK motif adopts a helix-loop-helix structure in which two alpha-helices are arranged in an anti-parallel fashion and the loop protrudes out of the frame to facilitate the formation of hairpin-like beta-sheet with neighboring loops. The conserved tetrapeptide T-P-L-H (6–9) forms a closed curve starting at the first alpha-helix of the ANK [79]. Hydrogen bonds between the threonine hydroxyl groups and the histidine imidazolic ring contribute to ANK stability. The Val/Ile-Val-XXX (hypophilic)-Leu/Val-Leu-Leu motif (positions 17–22) located in second alpha-helix stabilize the overall framework of an ANK protein. Glycine residues are stored at position 4 located in the second beta sheet, and at 13 and 25 at the alpha-helix ending [80].

ANK is a motif found in abundance in eukaryotic species, but little is known about the presence of ANK motif in viral proteins, except for species of the poxvirus genus that present ANK motifs in the terminal regions of the proteins involved in the ubiquitination process [81, 82]. A PSI-BLAST of the ANK domain was performed against poxvirus sequences, and it was possible to observe hits for the ANK motif, despite the low coverage presented.

The ORF wsv249 product was previously characterized as a ubiquitin ligase, acting as a key element in the modulation of protein abundance within cells through the ubiquitin-dependent proteolysis mechanism [83, 84]. The activity of ubiquitin-ligase is governed by the RING-H2 domain. The presence of ANK in conjunction with RING-H2 reinforces the function of wsv249 in the modulation of WSSV host proteins.

## Conclusion

The alignment of variable sequences revealed that the most and least variable regions are wsv129 and wsv 249 respectively, and that most of the already sequenced isolates did not present insertion in the wsv461/wsv464 cluster. The different approaches used were efficient in generating three-dimensional models and identifying domains, which enabled proposing functions for the putative polypeptides produced by the ORFs wsv249, wsv463a, wsv477, wsv479, wsv492, wsv497. The results indicate that these proteins are possibly involved in mechanisms related to protein modulation/degradation, intracellular transport, endosome recombination and fusion events. In addition, through the analysis of the secondary structure and characterization of the VNTR regions, it was possible to suggest that the products encoded by the ORFs wsv129 and wsv178 have structural function and may be involved in the WSSV adaptive mechanisms.

Considering that ORFs wsv463a, wsv479, wsv492 and wsv497 occur in a small number of WSSV isolates, their functions are not essential for the WSSV infection, or are being supplied by the cellular metabolism of the host. On the other hand, considering that wsv129, wsv178, wsv249 and wssv477 occur in all WSSV isolates and that sequence variations do not compromise the protein frame, their functions related to structural/packaging (wsv129, wsv178, wssv477) or in ubiquitination processes (wsv249) are possibly essential for viral replication and maintenance, and can be adaptive.

## Additional files

Additional file 1: Verify3D evaluation of protein models (DOCX 16 kb)

Additional file 2: Quality scores of predicted model of Formin Homology 2 domain (FH2). (A) Global QMEAN scores generated by Swiss-Model; (B) Ramachandran plots generated by pyRAMA; (C) Molprobity score. (PDF 1607 kb)

Additional file 3: Quality scores of the RNA recognition motif predicted model. (A) Global QMEAN scores generated by Swiss-Model; (B) Ramachandran plots generated by pyRAMA; (C) Molprobity score. (PDF 1496 kb)

Additional file 4: Quality scores of the XPD Helicase (wsv479) predicted model. (A) Global QMEAN scores generated by Swiss-Model; (B) Ramachandran plots generated by pyRAMA; (C) Molprobity score. (PDF 1582 kb)

Additional file 5: Quality scores of the XPD Helicase (wsv497) predicted model. (A) Global QMEAN scores generated by Swiss-Model; (B) Ramachandran plots generated by pyRAMA; (C) Molprobity score. (PDF 1611 kb)

Additional file 6: Quality scores of the HA2 hemagglutinin predicted model. (A) Global QMEAN scores generated by Swiss-Model; (B) Ramachandran plots generated by pyRAMA; (C) Molprobity score. (PDF 1495 kb)

Additional file 7: Quality scores of the Ankyrin repeat domain (ANK) predicted model. (A) Global QMEAN scores generated by Swiss-Model; (B) Ramachandran plots generated by pyRAMA; (C) Molprobity score. (PDF 1557 kb)

Additional file 8: Quality scores of the RING-H2 domain predicted model. (A) Global QMEAN scores generated by Swiss-Model; (B) Ramachandran plots generated by pyRAMA; (C) Molprobity score. (PDF 1469 kb)

## Abbreviations

ANK: Ankyrim Repeat
CDD: Conserved Domains Database
ELM: Eukaryotic Linear Motif
FH1: Formin Homology 1
FH2: Formin Homology 2
FHOD1: FH1/FH2 domain-containing protein 1
HA: Hemagglutinin
HIV: Human Immunodeficiency Virus
hnRNP A1: Heterogeneous Nuclear Ribonucleoprotein A1
ICP: Infected Cell Protein; mDia1: Mammalian Diaphanous 1
miR-7: MicroRNA 7
NER: Nuclear Excision Repair
NLS: Nuclear Localization Signal
NPC: Nuclear Pore Complex
ORF: Open Reading Frame
PDB: Protein Data Bank
RNF4: RING Finger Protein 4
RNP: Ribonucleoprotein
RRM: RNA Recognition Motif
RU: Repeat Units
TFIIH: Transcription Factor IIH
U1 snRNP: U1 small nuclear ribonucleoprotein
UIM: Ubiquitin-Interacting Motif
VNTR: Variable Number Tandem Repeat
VP9: Viral Protein 9
WSSV: White Spot Syndrome Virus
XPD: Xeroderma Pigmentosum Group D
