# Supplementary material for: Characterization of putative proteins encoded by variable ORFs in white spot syndrome virus genome
Source: BMC Struct Biol. 2019 Apr 18;19:8. doi: 10.1186/s12900-019-0106-y (PMC6474068; doi:10.1186/s12900-019-0106-y)
Supplement: Supplementary file 1 — Verify3D evaluation of protein models (DOCX 16 kb) [file 12900_2019_106_MOESM1_ESM.docx]

Additional file 1 - Verify3D evaluation of protein models.

| Model | Amino acid length | Percentage of amino acids that have reached acceptable 3D-1D score* |
| --- | --- | --- |
| Wsv249(ANK) | 208 | 75.48% |
| Wsv249(RING) | 56 | 26.79% |
| Wsv463a(FH2) | 339 | 37.17% |
| Wsv477(RRM) | 71 | 36.62% |
| Wsv479 | 418 | 53.35% |
| Wsv492 | 92 | 0.00% |
| Wsv497 | 481 | 34.72% |

*At least 80% of the amino acids have scored >= 0.2 in the 3D/1D profile.
