# Supplementary material for: Characterization of putative proteins encoded by variable ORFs in white spot syndrome virus genome
Source: BMC Struct Biol. 2019 Apr 18;19:8. doi: 10.1186/s12900-019-0106-y (PMC6474068; doi:10.1186/s12900-019-0106-y)
Supplement: Supplementary file 2 — Quality scores of predicted model of Formin Homology 2 domain (FH2). (A) Global QMEAN scores generated by Swiss-Model; (B) Ramachandran plots generated by pyRAMA; (C) Molprobity score. (PDF 1607 kb) [file 12900_2019_106_MOESM2_ESM.pdf]

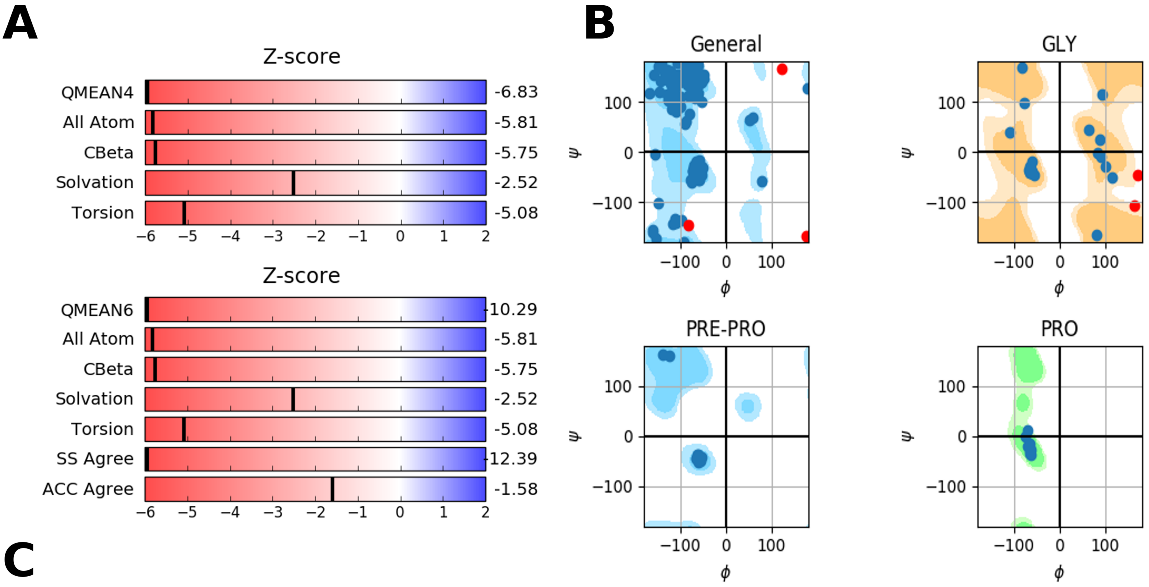

C

WSV463a_Formin_Homology_2_(FH2)			
Clashscore, all atoms:	103.57	0th percentile* (N=1784, all resolutions)	
Clashscore is the number of serious steric overlaps (> 0.4 Å) per 1000 atoms.			
Poor rotamers	7	2.40%	Goal: <0.3%
Favored rotamers	266	91.10%	Goal: >98%
Ramachandran outliers	5	1.48%	Goal: <0.05%
Ramachandran favored	309	91.69%	Goal: >98%
MolProbity score^	3.26	14th percentile* (N=27675, 0Å - 99Å)	
Cβ deviations >0.25Å	3	0.96%	Goal: 0
Bad bonds:	0 / 2663	0.00%	Goal: 0%
Bad angles:	49 / 3594	1.36%	Goal: <0.1%
Cis Prolines:	0 / 11	0.00%	Expected: ≤1 per chain, or ≤5%

Additional File 2
