# Supplementary material for: Characterization of putative proteins encoded by variable ORFs in white spot syndrome virus genome
Source: BMC Struct Biol. 2019 Apr 18;19:8. doi: 10.1186/s12900-019-0106-y (PMC6474068; doi:10.1186/s12900-019-0106-y)
Supplement: Supplementary file 3 — Quality scores of the RNA recognition motif predicted model. (A) Global QMEAN scores generated by Swiss-Model; (B) Ramachandran plots generated by pyRAMA; (C) Molprobity score. (PDF 1496 kb) [file 12900_2019_106_MOESM3_ESM.pdf]

A

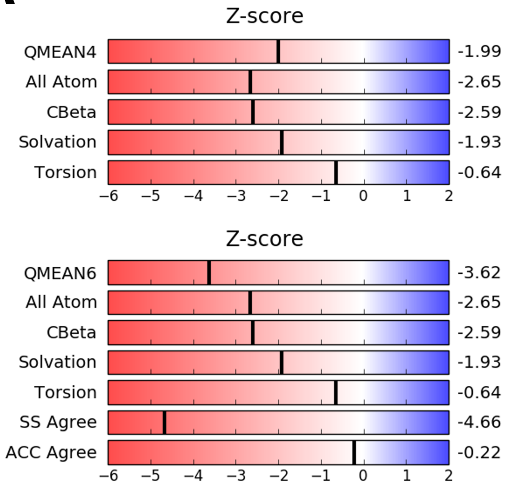

B

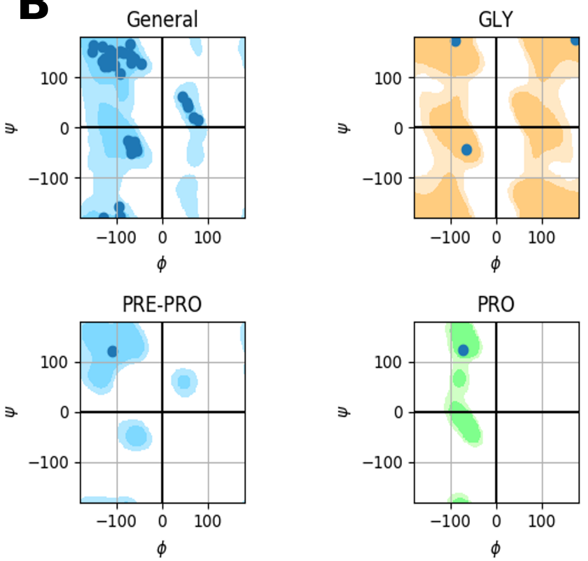

C

WSV477(ORF23)_RNA_Recognition_Motif_(RRM)			
Clashscore, all atoms:	94.83	0th percentile* (N=1784, all resolutions)	
Clashscore is the number of serious steric overlaps (> 0.4 Å) per 1000 atoms.			
Poor rotamers	3	4.69%	Goal: <0.3%
Favored rotamers	56	87.50%	Goal: >98%
Ramachandran outliers	0	0.00%	Goal: <0.05%
Ramachandran favored	67	97.10%	Goal: >98%
MolProbity score^	3.11	19th percentile* (N=27675, 0Å - 99Å)	
Cβ deviations >0.25Å	0	0.00%	Goal: 0
Bad bonds:	0 / 606	0.00%	Goal: 0%
Bad angles:	11 / 819	1.34%	Goal: <0.1%
Cis Prolines:	0 / 1	0.00%	Expected: ≤1 per chain, or ≤5%

Additional File 3
