# Supplementary material for: Characterization of putative proteins encoded by variable ORFs in white spot syndrome virus genome
Source: BMC Struct Biol. 2019 Apr 18;19:8. doi: 10.1186/s12900-019-0106-y (PMC6474068; doi:10.1186/s12900-019-0106-y)
Supplement: Supplementary file 4 — Quality scores of the XPD Helicase (wsv479) predicted model. (A) Global QMEAN scores generated by Swiss-Model; (B) Ramachandran plots generated by pyRAMA; (C) Molprobity score. (PDF 1582 kb) [file 12900_2019_106_MOESM4_ESM.pdf]

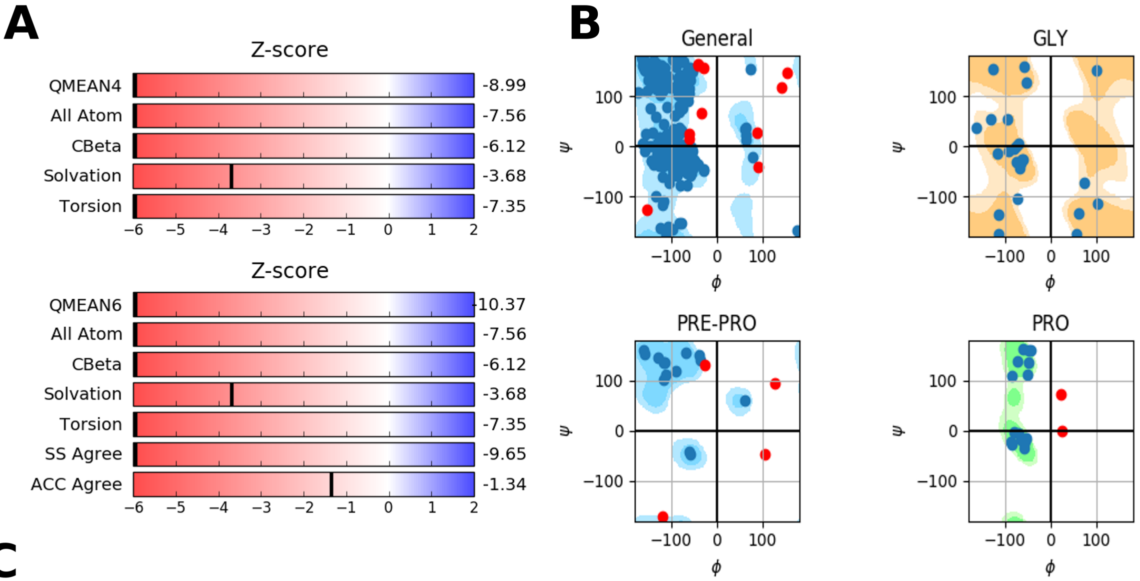

C

WSV479_XPD_Helicase			
Clashscore, all atoms:	10.8	67th percentile* (N=1784, all resolutions)	
Clashscore is the number of serious steric overlaps (> 0.4 Å) per 1000 atoms.			
Poor rotamers	13	3.48%	Goal: <0.3%
Favored rotamers	326	87.17%	Goal: >98%
Ramachandran outliers	22	5.29%	Goal: <0.05%
Ramachandran favored	337	81.01%	Goal: >98%
MolProbity score^	2.68	37th percentile* (N=27675, 0Å - 99Å)	
Cβ deviations >0.25Å	16	4.07%	Goal: 0
Bad bonds:	1 / 3411	0.03%	Goal: 0%
Bad angles:	83 / 4604	1.80%	Goal: <0.1%
Cis Prolines:	0 / 18	0.00%	Expected: ≤1 per chain, or ≤5%
Cis nonProlines:	2 / 399	0.50%	Goal: <0.05%
Twisted Peptides	6 / 417	1.44%	Goal: 0

Additional File 4
