# Supplementary material for: Characterization of putative proteins encoded by variable ORFs in white spot syndrome virus genome
Source: BMC Struct Biol. 2019 Apr 18;19:8. doi: 10.1186/s12900-019-0106-y (PMC6474068; doi:10.1186/s12900-019-0106-y)
Supplement: Supplementary file 5 — Quality scores of the XPD Helicase (wsv497) predicted model. (A) Global QMEAN scores generated by Swiss-Model; (B) Ramachandran plots generated by pyRAMA; (C) Molprobity score. (PDF 1611 kb) [file 12900_2019_106_MOESM5_ESM.pdf]

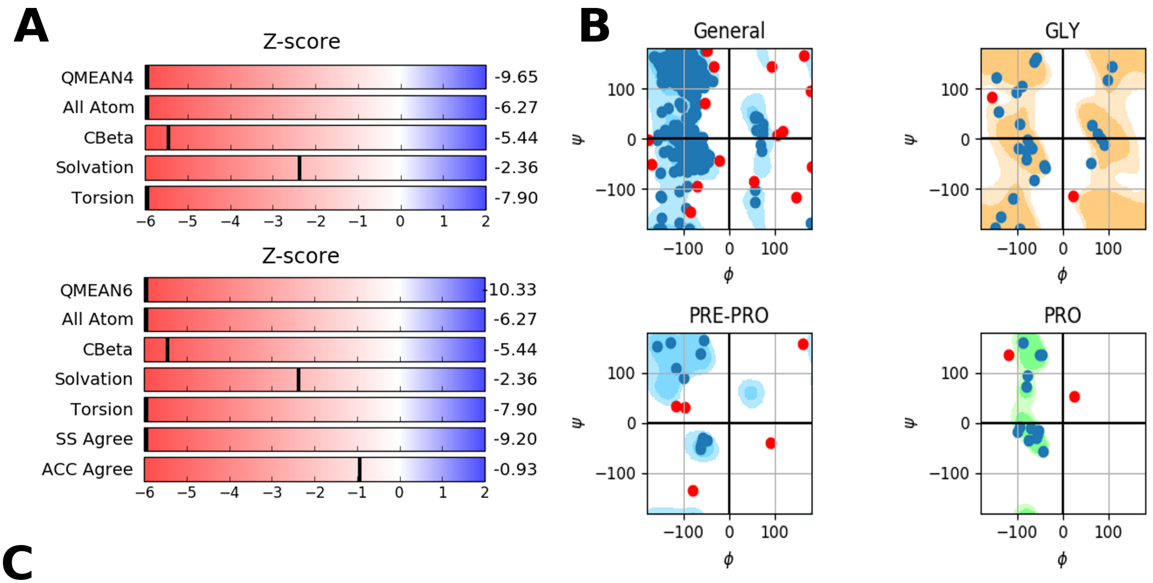

C

WSV497_XPD_Helicase			
Clashscore, all atoms:	2.97	98th percentile* (N=1784, all resolutions)	
Clashscore is the number of serious steric overlaps (> 0.4 Å) per 1000 atoms.			
Poor rotamers	6	1.38%	Goal: <0.3%
Favored rotamers	398	91.49%	Goal: >98%
Ramachandran outliers	26	5.43%	Goal: <0.05%
Ramachandran favored	388	81.00%	Goal: >98%
MolProbity score^	1.92	80th percentile* (N=27675, 0Å - 99Å)	
Cβ deviations >0.25Å	8	1.77%	Goal: 0
Bad bonds:	0 / 3938	0.00%	Goal: 0%
Bad angles:	66 / 5298	1.25%	Goal: <0.1%
Cis Prolines:	0 / 16	0.00%	Expected: ≤1 per chain, or ≤5%
Cis nonProlines:	8 / 464	1.72%	Goal: <0.05%
Twisted Peptides	2 / 480	0.42%	Goal: 0

Additional File 5
