# Supplementary material for: Characterization of putative proteins encoded by variable ORFs in white spot syndrome virus genome
Source: BMC Struct Biol. 2019 Apr 18;19:8. doi: 10.1186/s12900-019-0106-y (PMC6474068; doi:10.1186/s12900-019-0106-y)
Supplement: Supplementary file 6 — Quality scores of the HA2 hemagglutinin predicted model. (A) Global QMEAN scores generated by Swiss-Model; (B) Ramachandran plots generated by pyRAMA; (C) Molprobity score. (PDF 1495 kb) [file 12900_2019_106_MOESM6_ESM.pdf]

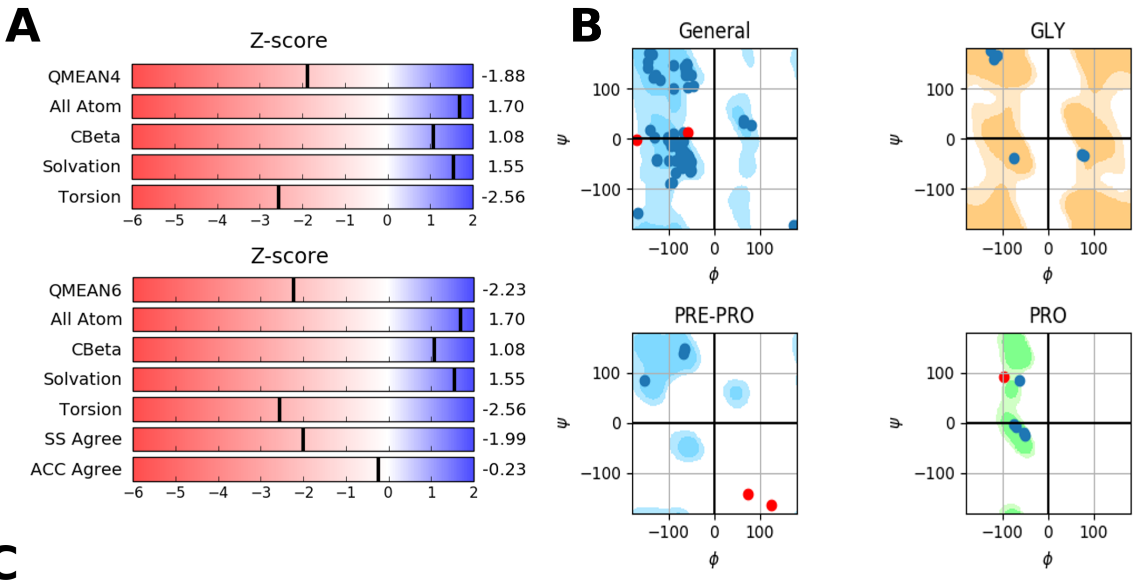

C

WSV492_Hemagglutinin_(HA2)			
Clashscore, all atoms:	6.9	87th percentile* (N=1784, all resolutions)	
Clashscore is the number of serious steric overlaps (> 0.4 Å) per 1000 atoms.			
Poor rotamers	12	4.71%	Goal: <0.3%
Favored rotamers	227	89.02%	Goal: >98%
Ramachandran outliers	7	2.59%	Goal: <0.05%
Ramachandran favored	249	92.22%	Goal: >98%
MolProbity score^	2.37	55th percentile* (N=27675, 0Å - 99Å)	
Cβ deviations >0.25Å	8	2.96%	Goal: 0
Bad bonds:	1 / 2349	0.04%	Goal: 0%
Bad angles:	46 / 3138	1.47%	Goal: <0.1%
Cis Prolines:	0 / 6	0.00%	Expected: ≤1 per chain, or ≤5%

Additional File 6
