# Supplementary material for: Characterization of putative proteins encoded by variable ORFs in white spot syndrome virus genome
Source: BMC Struct Biol. 2019 Apr 18;19:8. doi: 10.1186/s12900-019-0106-y (PMC6474068; doi:10.1186/s12900-019-0106-y)
Supplement: Supplementary file 7 — Quality scores of the Ankyrin repeat domain (ANK) predicted model. (A) Global QMEAN scores generated by Swiss-Model; (B) Ramachandran plots generated by pyRAMA; (C) Molprobity score. (PDF 1557 kb) [file 12900_2019_106_MOESM7_ESM.pdf]

A

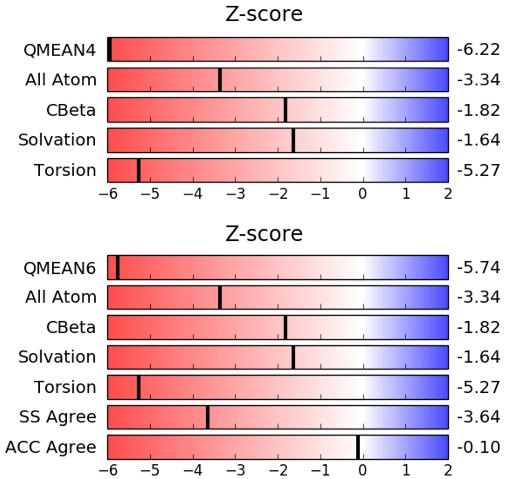

B

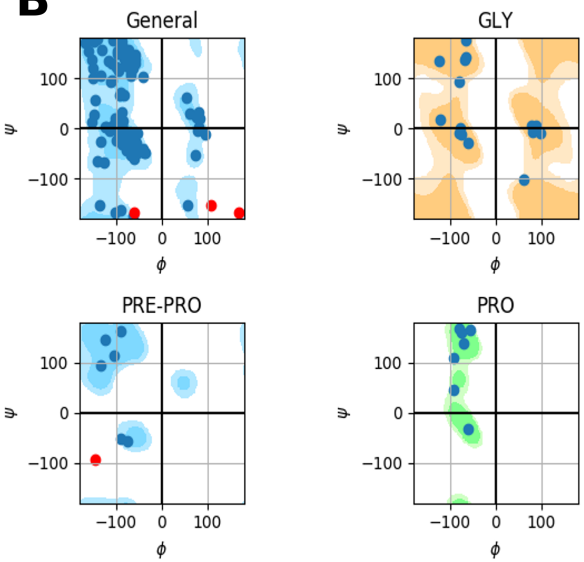

C

WSV249(ORF125)_Ankyrin_Repeat_(ANK)			
Clashscore, all atoms:	9.45	74th percentile* (N=1784, all resolutions)	
Clashscore is the number of serious steric overlaps (> 0.4 Å) per 1000 atoms.			
Poor rotamers	5	2.82%	Goal: <0.3%
Favored rotamers	164	92.66%	Goal: >98%
Ramachandran outliers	5	2.43%	Goal: <0.05%
Ramachandran favored	170	82.52%	Goal: >98%
MolProbity score^	2.54	45th percentile* (N=27675, 0Å - 99Å)	
Cβ deviations >0.25Å	5	2.60%	Goal: 0
Bad bonds:	0 / 1676	0.00%	Goal: 0%
Bad angles:	28 / 2259	1.24%	Goal: <0.1%
Cis Prolines:	0 / 7	0.00%	Expected: ≤1 per chain, or ≤5%
Cis nonProlines:	1 / 200	0.50%	Goal: <0.05%
Twisted Peptides	2 / 207	0.97%	Goal: 0

Additional File 7
