# Supplementary material for: Characterization of putative proteins encoded by variable ORFs in white spot syndrome virus genome
Source: BMC Struct Biol. 2019 Apr 18;19:8. doi: 10.1186/s12900-019-0106-y (PMC6474068; doi:10.1186/s12900-019-0106-y)
Supplement: Supplementary file 8 — Quality scores of the RING-H2 domain predicted model. (A) Global QMEAN scores generated by Swiss-Model; (B) Ramachandran plots generated by pyRAMA; (C) Molprobity score. (PDF 1469 kb) [file 12900_2019_106_MOESM8_ESM.pdf]

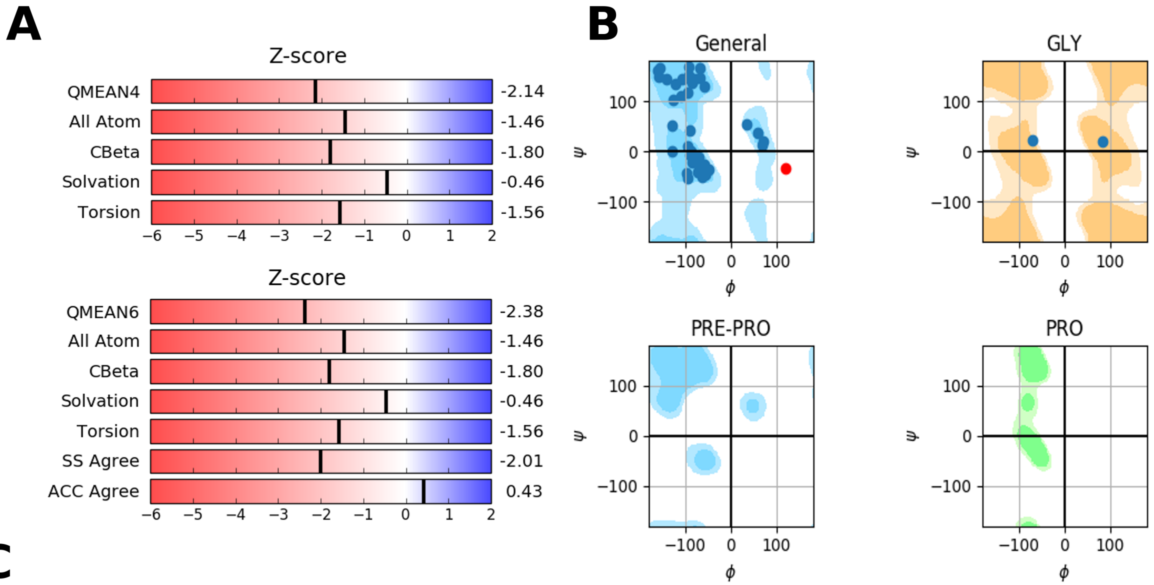

C

WSV249(ORF125)_RING-H2			
Clashscore, all atoms:	2.24	99th percentile* (N=1784, all resolutions)	
Clashscore is the number of serious steric overlaps (> 0.4 Å) per 1000 atoms.			
Poor rotamers	1	1.89%	Goal: <0.3%
Favored rotamers	49	92.45%	Goal: >98%
Ramachandran outliers	1	1.85%	Goal: <0.05%
Ramachandran favored	50	92.59%	Goal: >98%
MolProbity score^	1.67	90th percentile* (N=27675, 0Å - 99Å)	
Cβ deviations >0.25Å	1	1.85%	Goal: 0
Bad bonds:	0 / 451	0.00%	Goal: 0%
Bad angles:	11 / 603	1.82%	Goal: <0.1%
Cis Prolines:	0 / 0	0.00%	Expected: ≤1 per chain, or ≤5%

Additional File 8
